# 77232 The role of the bromodomain and extra-terminal motif (BET) family of proteins in head and neck cancer tumorigenic phenotype

**DOI:** 10.1017/cts.2021.415

**Published:** 2021-03-30

**Authors:** Reilly A. Sample, Rachel Paolini

**Affiliations:** Washington University in St. Louis School of Medicine

## Abstract

ABSTRACT IMPACT: Understanding the biology underpinning head and neck cancer invasion and metastasis could lead to novel targeted therapies that are both effective and tolerable for patients with this debilitating disease. OBJECTIVES/GOALS: The bromodomain and extra-terminal (BET) family of epigenetic regulators has been implicated in the tumorigenesis of various cancers. In head and neck squamous cell carcinoma (HNSCC), the majority of morbidity is due to invasion and metastasis, so there is special interest in understanding the development of these phenotypes in HNSCC cells. METHODS/STUDY POPULATION: Stable SCC9 knockdown cell lines were generated by infecting cells with a lentivirus encoding Cas9-KRAB and a lentivirus encoding gRNA targeting each of the candidate BET proteins: BRD2, BRD3, BRD4, and BRDT. Knockdown was confirmed by qRT-PCR. Next, standard assays for proliferation (CellTiter-glo), invasion (Matrigel), and migration (scratch-wound healing assay) were performed for all candidate knockdowns and compared to a non-target control. RESULTS/ANTICIPATED RESULTS: Proliferation assay results revealed that BRD4 knockdown had a significant negative effect on the proliferative capacity of SCC9 cells in vitro. Similarly, BRD4 knockdown SCC9 cell lines were less invasive and less migratory. Interestingly, knockdown of BRD2, BRD3, and BRDT had no effect on proliferation, invasiveness, or migration. DISCUSSION/SIGNIFICANCE OF FINDINGS: We have identified BRD4 as a key driver of the HNSCC tumorigenic phenotype. In the future, we plan to investigate the role of JQ1, a pan-BET inhibitor, on HNSCC cell phenotypes. Additionally, we will identify the downstream targets of BRD4, which may serve as potential therapeutic targets for both HNSCC as well as other cancers more broadly.

